# Attitudes Toward Providing Open Access for Use of Biospecimens and Health Records: A Cross-Sectional Study from Jordan

**DOI:** 10.2147/PPA.S402769

**Published:** 2023-03-28

**Authors:** Kamal M Al-Shami, Wesam S Ahmed, Karem H Alzoubi

**Affiliations:** 1 Department of Clinical Pharmacy, Faculty of Pharmacy, Jordan University of Science and Technology, Irbid, 22110, Jordan; 2 Faculty of Biosciences, University of Heidelberg, Heidelberg, 69120, Germany; 3 College of Health and Life Sciences, Hamad Bin Khalifa University, Qatar Foundation, Doha, Qatar

**Keywords:** biospecimens, health records, Jordan, open access, blanket consent, clinical research

## Abstract

**Purpose:**

Biospecimen repositories and big data generated from clinical research are critically important in advancing patient-centered healthcare. However, ethical considerations arising from reusing clinical samples and health records for subsequent research pose a hurdle for big-data health research. This study aims to assess the public’s opinions in Jordan toward providing blanket consent for using biospecimens and health records in research.

**Participants and Methods:**

A cross-sectional study utilizing a self-reported questionnaire was carried out in different cities in Jordan, targeting adult participants. Outcome variables included awareness of clinical research, participation in clinical research, and opinions toward providing open access to clinical samples and records for research purposes. Descriptive analysis was utilized for reporting the outcome as frequency (percentages) out of the total responses. Univariate and multivariate logistic regression were used to investigate the association between independent variables and the outcome of interest.

**Results:**

A total of 1033 eligible participants completed the questionnaire. Although the majority (90%) were aware of clinical research, only 24% have ever participated in this type of research. About half (51%) agreed on providing blanket consent for the use of clinical samples, while a lower percentage (43%) agreed on providing open access to their health records. Privacy concerns and lack of trust in the researcher were cited as major barriers to providing blanket consent. Participation in clinical research and having health insurance were predictors for providing open access to clinical samples and records.

**Conclusion:**

The lack of public trust in Jordan toward data privacy is evident from this study. Therefore, a governance framework is needed to raise and maintain the public’s trust in big-data research that warrants the future reuse of clinical samples and records. As such, the current study provides valuable insights that will inform the design of effective consent protocols required in data-intensive health research.

## Introduction

Clinical research is a fundamental tool for understanding diseases and designing preventive and therapeutic strategies.^1–3^ Jordan is an Arab country in the Middle East and North Africa (MENA) region with progressive research agenda.^1^,^4^,^5^ The county is ranked among the top countries in the Arab region based on clinical studies per capita (Table 1). Additionally, it was the first Arab country to enact clinical research regulations.^6^ The country is known for its flourished pharmaceutical industry, which exports its products to more than 60 countries globally, making it the second-largest exporting industry in the country.^7–9^ Moreover, Jordan hosts several clinical study centers, some of which conduct clinical trials on behalf of international pharmaceutical companies.^10^ The country also has a well-established cancer biobank, the King Hussein Cancer Center Biobank, which is the first ISO-accredited cancer biobank from a diverse ethnic MENA population.^11^

**Table 1:** Comparison Between the Number of Clinical Studies in the Arab MENA Region

Country	Number of Clinical Studies^†^	Population (Millions)^‡^	Clinical Studies per 100,000 Capita
Lebanon	327	6.82	4.79
Tunisia	187	11.82	1.58
Qatar	52	2.88	1.81
Jordan	156	10.20	1.53
Egypt	1567	102.33	1.53
Kuwait	62	4.27	1.45
UAE	139	9.89	1.41
KSA	370	34.81	1.06
Oman	38	5.12	0.74
Morocco	65	36.91	0.18
Syria	25	17.50	0.14
Algeria	51	43.85	0.12
Iraq	35	40.22	0.09
Libya	4	6.87	0.06
Yemen	2	29.82	0.01

**Notes**: ^†^Retrieved from 2022 US NIH registry of clinical trials: ClinicalTrials.gov. ^‡^Retrieved from 2022 World Bank: https://data.worldbank.org/.

Establishing clinical sample repositories and the digitalization of medical health records, accompanied by the ease of communication and the over-sees research collaboration, has opened the door for big-data clinical research that, if utilized effectively, can provide the premise for precision medicine and patient-centered healthcare. However, the need to obtain repeated patient consent for subsequent clinical research creates a hurdle for researchers and delays the commencement of research projects. Therefore, consenting models, such as “blanket consent” and “broad consent”, have been adopted by several biobank projects to balance the interest of the donors and researchers.^12^,^13^ Blanket consent implies providing open access to a broad range of future studies without restriction.^14^,^15^ This is slightly different from broad consent, which means consenting to a framework of future research subjected to limited predefined restrictions.^16^,^17^ Hence, given the active clinical research agenda in country and the importance of having a robust consenting model for big-data clinical research, the current study explores Jordan’s public views toward providing blanket consent for use of biospecimens and health records, aiming to overcome barriers and enhance positive attitudes toward providing the same.

To achieve this, a self-administered questionnaire was distributed to eligible participants. Results showed a lack of social license to reuse medical samples and records in clinical research. Participants were more stringent on providing access to health records than to clinical samples. To encourage social license, there is a need to identify conditions that lead to public distrust, and to establish a governance framework that considers patient and public views, needs, values, and interests.

## Methods

### Study Design and Participants

A questionnaire survey was carefully constructed after reviewing similar work from the region.^18–22^ The survey was distributed by experienced interviewers to eligible participants in public places in different cities in Jordan, such as Amman, Zarqa, Karak, Irbid, and Mafraq, from Feb 2019 to March 2019 using a convenience sampling approach. Eligibility criteria included autonomy, competency, an age of more than 18, and the ability to read and understand the Arabic language. The questionnaire was distributed in Arabic since it is the native language of the country. Informed oral consent was obtained from all participants after providing them with a detailed description of the study as well as contact information should they decide to withdraw or have any concerns regarding the survey. The participants were then provided with a brief description of clinical research, its scope, the consent process, and its different forms, including the definition of “open access”. Response formats utilized in the three-sectioned questionnaire included multiple-choice, “Yes” or “No”, multiple check boxes, and Likert scale items. The first section solicited sociodemographic information from respondents such as age, gender, marital status, nationality, education level, health insurance status, and whether they have any chronic medical conditions. The second section assessed participants’ awareness of “clinical research”, whether they have previously participated in clinical research, and their willingness to participate in clinical research in the future. The last section solicited participants’ views toward providing open access to their biospecimens and health records for research purposes. The study was verified by the Institutional Review Board (IRB) committee of Jordan University of Science and Technology (Ref# 38/117/2018) with no consent form requirement.

### Data Analysis

Data were analyzed using statistical package for social science (SPSS) version 22 (SPSS Inc., Chicago, IL, USA). Power analysis was performed, ensuring power is more than 80%. Descriptive analysis was performed to summarize the data by reporting the frequency (percentages) out of the total responses approximated to two decimals in the tables and zero decimals in the text. Answers to the follow up, open ended questions were mapped into general categories and reported as frequency (percentages) out of the total answers. Univariate and multivariate logistic regression was performed to screen for variables associated with hesitancy toward providing open access to clinical samples and medical records. Following the univariate logistic regression analysis, any variable that was significant on the single predictor level (P-value <0.25) was entered into the multivariate logistic regression analysis to explore predictors significantly and independently associated with hesitancy toward providing blanket consent. Odds ratio were calculated to estimate the effect of each predictor on the outcome. A P value of ≤ 0.05 was considered statistically significant. Figures were prepared using Microsoft Excel 13.

## Results

### Demographics

The sociodemographic characteristics are summarized in Table 2. A total of 1033 participants completed the survey. The response rate was 85%. Approximately half of the participants (48%) were less than 24 years old. There was an almost equal distribution of male to female participants (50%, n=512, 50%, n=521). Most participants were Jordanians (84%, n=865), single (75%, n=777), and have received a diploma or a higher education degree (84%, n=869). Most were medically insured (67%, n=696) and had no chronic clinical conditions (90%, n=934).

**Table 2:** Sociodemographic Characteristics of the Participants (n=1033)

Variable	N	%
Gender		
Male	512	49.56
Female	521	50.44
Age		
< 24 years old	497	48.11
24–35 years old	376	36.40
> 35 years old	160	15.49
Nationality		
Jordanian	865	83.74
Non-Jordanian	168	16.26
Marital status		
Single	777	75.22
Married	239	23.14
Divorced	13	1.26
Widowed	4	0.39
Level of education		
Less than diploma	164	15.88
Diploma or higher education	869	84.12
Have health insurance		
Yes	696	67.38
No	337	32.62
Have a chronic medical condition		
Yes	99	9.58
No	934	90.42

### Awareness and Participation in Clinical Research

**Figure 1:**
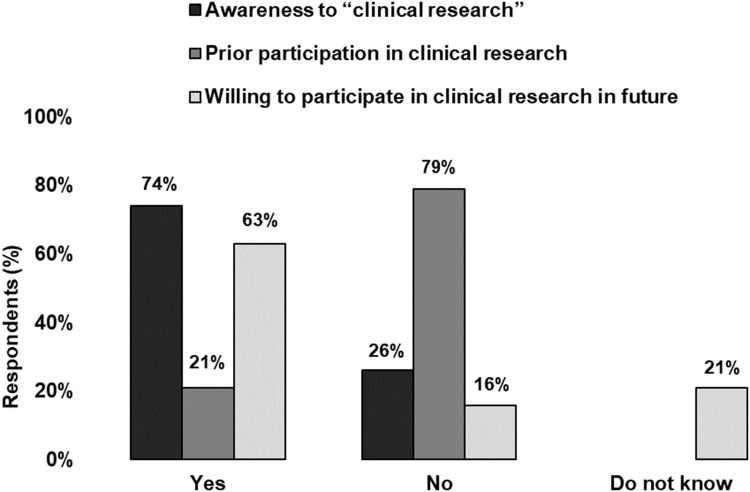
Awareness and participation in clinical research. Bar graph showing the percentage of participants who reported being aware of “clinical research” (black), who have previously participated in clinical research (dark gray), and who are willing to participate in clinical research in the future (light gray).

Most participants (74%, n=768) reported understanding the “clinical research” terminology. A minority reported (21%, n=212) prior participation in clinical research. However, the majority (63%, n=650) were willing to participate if they were invited in the future (Figure 1).

### Attitudes Toward Providing Open Access to Clinical Samples and Health Records

Around 51% (n=528) and 44% (n=454) of the participants agreed on providing blanket consent for the use of clinical samples (blood) and health records, respectively. Participants’ reasons for refusing to provide blanket consent are summarized in Table 3 and Table 4. Results from univariate and multivariate logistic regression revealed predictors associated with participants’ hesitancy toward giving open access to blood samples and health records (Table 5 and Table 6).

**Table 3:** Participants Reported Barriers to Providing Blanket Consent for the Use of Blood Samples (n=505, 48.89%)

Barrier	N	%
May reveal sensitive data about me, and I want my privacy to be respected	282	27.30
I do not trust researchers, so I need to be informed how my blood will be used before providing the consent	161	15.59
I have a blood disease	15	1.45
It is not ethical	12	1.16
Other reasons	35	3.39

**Table 4:** Participants Reported Barriers for Providing Blanket Consent for the Use of Health Records (n=579, 56.05%)

Barrier	N	%
May reveal sensitive data about me, and I want my privacy to be respected	293	28.36
I do not trust researchers, so I need to be informed how my health record will be used before providing the consent	131	12.68
It is not ethical	14	1.35
Other reasons	47	4.55
No answer	94	9.10

**Table 5:** Variables Associated with Hesitancy Toward Providing Open Access to Blood Samples

Variable	Provide Open Access to Blood Samples [0: Yes (51.11%, n=528), 1: No (48.89%, n=505)]			
	Univariable Logistic Regression		Multivariable Logistic Regression	
	OR (95% CI)	P value	OR (95% CI)	P value
Gender				
Male	Reference			
Female	1.33 (1.04–1.70)	0.0228^†^	1.25 (0.97–1.62)	0.0958
Age (years old)				
< 24	Reference			
24–35	0.80 (0.61–1.05)	0.1074^†^	0.89 (0.6583–1.195)	0.4297
> 35 years old	0.69 (0.482–0.99)	0.0437^†^	0.74 (0.49–1.12)	0.1417
Nationality				
Jordanian	Reference			
Non-Jordanian	1.03 (0.74–1.43)	0.8833	----	----
Marital status				
Single	Reference			
Married	0.73 (0.55–0.99)	0.0367^†^	0.83 (0.59–1.16)	0.2646
Divorced	0.60 (0.181–1.83)	0.3809	----	----
Widowed	0.97 (0.12–8.09)	0.9734	----	----
Level of education				
Before higher education	Reference			
Higher education	1.43 (1.02–2.01)	0.0387^†^	1.32 (0.92–1.90)	0.1337
Have health insurance				
Yes	Reference			
No	0.65 (0.42–0.98)	0.0435^†^	0.57 (0.37–0.88)	0.0113*
Have a chronic medical condition				
Yes	Reference			
No	1.04 (0.80–1.35)	0.7650	----	----
Awareness of “clinical research”				
Yes	Reference			
No	0.97 (0.73–1.28)	0.8252	----	----
Prior participation in clinical research				
Yes	Reference			
No	0.97 (0.72–1.31)	0.8340	----	----
Willing to participate in clinical research in the future				
Yes	Reference			
No	1.57 (1.11–2.22)	0.0108^†^	1.76 (1.24–2.52)	0.0019**
Do not know	1.35 (0.99–1.83)	0.0573^†^	1.34 (0.98–1.84)	0.0639

**Note**: ^†^Eligible for entry into multiple logistic regression analysis; *,**Refer to a P value of less than 0.05 and 0.005, respectively.

**Table 6:** Variables Associated with Hesitancy Toward Providing Open Access to Health Records

Variable	Provide Open Access to Health Records [0: Yes (43.95%, n=454), 1: No (56.05%, n=579)]			
	Univariable Logistic Regression		Multivariable Logistic Regression	
	OR (95% CI)	P value	OR (95% CI)	P value
Gender				
Male	Reference			
Female	1.61 (1.25–2.06)	0.0002^†^	1.43 (1.10–1.86)	0.0086*
Age (years old)				
< 24	Reference			
24–35	0.76 (0.58–0.99)	0.0430^†^	0.84 (0.62–1.14)	0.2552
> 35 years old	0.59 (0.41–0.84)	0.0038^†^	0.65 (0.44–0.98)	0.0415*
Nationality				
Jordanian	Reference			
Non-Jordanian	0.79 (0.57–1.10)	0.1660^†^	0.85 (0.60–1.20)	0.3423
Marital status				
Single	Reference			
Married	0.75 (0.56–0.997)	0.0474^†^	0.91 (0.65–1.28)	0.5889
Divorced	0.62 (0.20–1.9)	0.3988	----	----
Widowed	0.73 (0.09–6.08)	0.7501	----	----
Level of education				
Before higher education	Reference			
Higher education	1.69 (1.21–2.37)	0.0023^†^	1.39 (0.97–1.99)	0.0774
Have health insurance				
Yes	Reference			
No	0.71 (0.46–1.08)	0.1111^†^	0.62 (0.40–0.97)	0.0401*
Have a chronic medical condition				
Yes	Reference			
No	0.81 (0.62–1.05)	0.1121^†^	0.95 (0.72–1.25)	0.7207
Awareness of “clinical research”				
Yes	Reference			
No	0.84 (0.63–1.11)	0.2209^†^	0.94 (0.70–1.27)	0.7016
Prior participation in clinical research				
Yes	Reference			
No	0.69 (0.50–0.94)	0.0189^†^	0.70 (0.50–0.96)	0.0267*
Willing to participate in clinical research in the future				
Yes	Reference			
No	0.97 (0.69–1.37)	0.8513	----	----
Do not know	1.098 (0.81–1.50)	0.5532	----	----

**Notes**: ^†^Eligible for entry into multiple logistic regression analysis; *Refers to P value of less than 0.05.

## Discussion

In this study, we investigated the public attitudes in Jordan toward providing open access to their clinical samples and health records for research purposes. Results indicated a public’s unease toward providing free access to medical samples and records, with more stringent attitudes toward providing indefinite access to health records than medical samples.

People older than 35 were more reluctant to provide blanket consent for the use of health records. This may be related to the social stigma that may arise if sensitive information is revealed about older participants, especially since older age is a risk factor for several diseases.^23^,^24^ Respecting one’s privacy was the most cited reason in our study for rejecting to provide open access to health records. For similar reasons, the female gender was associated with more reluctance to provide blanket consent for the use of health records, as women privacy is critically valued in the region.^25–27^ Moreover, health insurance was positively associated with the rejection of providing open access to blood samples and health records. Those who are medically insured may fear losing their insurance if some of their sensitive information were released.^28^,^29^

Most participants (90%) reported being aware of clinical research. This could be attributed to the high education level of the participants. After all, the country reportedly has the highest literacy rate in the Arab world.^30^,^31^ The country is also well known in the region for its pharmaceutical industry and has several clinical research centers scattered across its cities.^7–9^ As such, the familiarity with the “clinical research” in the country may be higher than in other countries in the region, for instance, in Lebanon, where only 45% reported recognizing the same terminology.^21^

Participation in research is an important aspect that may influence participants’ decision to provide blanket consent.^32–34^ Assessing participation in clinical research revealed that even though a minority (21%) have participated in clinical research, the majority (63%) were willing to participate in the future. This may suggest that most recruitments in the country occur in clinical settings and, therefore, the need for outreach strategies to recruit more of the general public, which is expected to reflect more accurately the research findings.^35^,^36^ Studies from other countries in the region, such as Qatar and Saudi Arabia, also showed that most participants (63% and 74%, respectively) had positive attitudes toward participation.^18^,^19^ More importantly, multivariate analysis revealed that willingness to participate in research was positively associated with attitudes toward providing blanket consent for the use of blood samples. A similar association was reported in previous studies.^34^,^37–39^ On the other hand, there was a positive association between prior participation in clinical research and the reluctance to provide open access for the use of health records. As such, it would seem that there is a need to modify the current communication strategies between researchers and participants and make the recruitment process as seemingly as possible to apparently enhance participants’ experience and elevate the public trust in researchers.^40–44^ After all, lack of trust in researchers was cited as the second most common barrier to providing open access to blood samples and health records in our study.

The consent process is a fundamental step in clinical research that involves human subjects.^45–47^ Almost half of the participants were willing to provide open access to their blood samples, while less than half agreed to provide open access to their health records. Fear of privacy being negatively affected was the most reported reason for rejecting to provide blanket consent, followed by a lack of trust in researchers. Indeed, other studies also cited privacy concerns as barriers.^39^,^48^ On the other hand, trust is a fundamental aspect of research and has been cited as a positive predictor for providing blanket consent.^34^,^37^,^38^ Therefore, strengthening the measures taken to preserve data confidentiality and informing the potential participants about these measures that protect their privacy could be effective strategies to enhance the public trust in researchers.

## Limitations

This study has some limitations that can be addressed and mitigated through future research. For instance, although the current study investigated the public attitude toward providing indefinite access to clinical samples and health records, it did not investigate the reasons that may contribute to the social acceptance of a blanket consent protocol. In addition, despite that, the recruitment process taking place in different places in other cities in Jordan, the sampling technique that was followed in this study is still a convenience sampling approach by definition, and the ability to infer generalizability from a convenience sample is still limited compared to some other sampling techniques such as random sampling.

## Conclusion

The study revealed a lack of social licensing for the indefinite use of biospecimens and health records in clinical research. Results from the current study shall inform the decision makers about the current public attitude toward integrating a blanket consent protocol and call for further research to explore elements that may contribute to the social acceptance of such protocol. In addition, factors contributing to the public unease toward providing blanket consent need to be addressed and mitigated through future research.

## Data Sharing Statement

The survey questionnaire will be provided upon reasonable request from the first authors.
